# Roles of No-Go RNA decay in the control of plant viruses and transposable elements

**DOI:** 10.1186/s12870-025-07463-0

**Published:** 2025-10-17

**Authors:** Benjamin Shone, Zhen Lei, Jungnam Cho

**Affiliations:** 1 Department of Biosciences, Durham University, Durham, DH1 3LE UK; 2 Department of Neuroscience, Yale University School of Medicine, New Haven, CT 06510 United States of America

**Keywords:** No-go RNA decay (NGD), Ribosome stalling, RNA quality control (RQC), Viral infection, Transposable element (TE), RNA-directed DNA methylation (RdDM)

## Abstract

Plant cells constantly face genetic invasions from both external and internal sources. Viruses and transgenes represent major external threats, while transposable elements (TEs) are endogenous sources of invasive DNA. The early recognition and activation of innate defence mechanisms are therefore critical for maintaining genome integrity. Emerging evidence suggests that foreign genetic elements are detected and processed by the ribosome-associated RNA quality control system, a key cellular pathway responsible for resolving aberrant transcripts with translation defects. One such pathway, known as No-Go RNA Decay (NGD), facilitates RNA cleavage and ribosome dissociation at stalled ribosomes. Genetic and biochemical studies indicate that NGD plays a crucial role in plant antiviral defence and TE regulation, positioning it as a potential first line of defence against invasive genetic elements. This review explores recent advances in plant NGD research, shedding light on the fundamental question of how cells distinguish self from non-self nucleic acids.

## Introduction

In eukaryotic cells, low quality mRNA transcripts are tightly monitored as they can result in protein synthesis errors and the production of non-functional or toxic proteins [3, 15, 18]. As a result, RNA quality control (RQC) pathways exist to mitigate such fatal cellular defects. In addition to its surveillance function in the cell, accumulating evidence suggests that RQC plays an important role in gene expression control [31]. The transcripts subject to the RQC pathways may possess various characteristics such as complex RNA secondary structure, damage by ribonucleases or reactive chemical species and rare codons [26, 49]. Current understanding describes three major RQC pathways, namely nonsense-mediated decay (NMD), non-stop decay (NSD), and no-go decay (NGD) (recently reviewed in Monaghan et al. [31]. NMD degrades mRNAs that contain premature stop codons to prevent the production of truncated and potentially deleterious proteins. Mediated by Up-frameshift protein 1, 2 and 3 (UPF1/2/3), NMD regulates plant gene expression as well as plant defence response [17, 40]. Conversely, NSD targets mRNA lacking a stop codon, preventing the formation of potentially detrimental elongated proteins. NSD is triggered by stalled ribosomes at the 3’ end of mRNA. As in NGD that will be detailed below, the Dom34-Hbs1 heterodimer complex is also associated with ribosome rescue in NSD.

NGD is triggered by stacks of stalled ribosomes and results in ribosome dissociation and degradation of mRNA [43, 44]. This process is largely mediated by the Dom34-Hbs1 complex, which is related and structurally akin to the eukaryotic release factors [50]. Previous studies in yeast have shown that endoribonucleolytic cleavage occurs within a stalled ribosome exit tunnel, exposing a hydroxyl group on the 5’ end of the truncated mRNA [34]. The endonuclease Cue2 has been strongly implicated as responsible for the cleavage of mRNA located within ribosome stall sites in yeast and may be conserved across eukaryotes, while it seems to be absent in plant species [8, 35]. The kinase tRNA ligase 1 (Trl1) then phosphorylates 5’-OH ends, producing 5’-monophosphate (P) required for cytoplasmic RNA decay [34]. Finally, Exoribonuclease 1 (XRN1) targets 5’P-RNA for RNA degradation, completing the NGD pathway [14].

In plants, while NMD has been well characterized [28], ribosome-coupled RQC has been relatively under-investigated, and its biological functions have just started to be unveiled. Importantly, recent studies highlighted that NGD plays a critical role particularly in controlling invasive nucleic acids, for example, those of viruses as well as transposable elements (TEs), mobile DNA sequences that share similarities in mobilisation and replication to viruses. This suggests that ribosome-associated process, mainly through NGD, might act as an innate defence pathway against invasive genetic elements, which is of fundamental importance to plants given their sessile lifestyle. This review highlights and discusses NGD controls viruses and TEs in plants.

## Main text

### Viral pathogen control

Viruses rely on host cell translational machinery to produce viral proteins necessary for their propagation. Therefore, from the hosts point of view, ribosomes are at the forefront to invading viruses. Given the distant evolutionary divergence, viral sequences are not as optimal for translation as endogenous mRNAs [27]. Consequently, viral RNAs tend to be more vulnerable to the host RNA degradation system, including NGD [41].

Orthologues of yeast Dom34 in other species are also known as Pelota, and in plants, Pelota is implicated in viral control. In Arabidopsis, for example, NGD can target specific motifs within turnip mosaic virus (TuMV), initiating Pelota-Hbs1-mediated cleavage of the TuMV RNA [10]. Specifically, Pelota has been shown to target the potyvirid G1-2A6-7 motif (e.g., GGAAAAAA) found across the potyvirid family, including TuMV [10]. In line with this observation, TuMV has also been shown to be suppressed by XRN4, a primary mRNA degrading enzyme in Arabidopsis and key factor in the NGD pathway [23]. This NGD-associated viral repression mechanism is of particular relevance in agriculture as potyvirid viruses are known to be responsible for approximately half of viral crop damage worldwide [52].

In a crop model, Sun et al. showed that overexpression of rice *Pelota*, *OsPelo*, resulted in reduced infectivity of southern rice black-streaked dwarf virus (SRBSDV), phytoreovirus RDV, and cytorhabdovirus RSMV in rice [47]. This seems to be because Pelota directly interacts with P7-1 tubules, preventing its formation in the host cells, which is essential for the movement and proliferation of SRBSDV [13, 47]. Intriguingly, the knockout mutants of *OsPelo* also led to a decrease in viral protein production. Since the levels of ribosomal proteins are reduced in these mutants and viruses exploit the hosts translational machinery for their own propagation, the decrease of viral proteins in the *ospelo* mutants might be due to a compromised ribosome homeostasis independent on the surveillance function of NGD.

Similar observations have been made regarding *Pelota* mediating begomovirus resistance. Within peppers, suppression of *CaPelota*, a homolog of *Pelota*, conferred resistance against the yellow curly leaf disease phenotype associated with begomovirus infection [19]. This has also been observed in tomatoes. In a study by Lapidot et al. suppression of the *Pelota* homolog conferred yellow curly leaf disease resistance in tomatoes [21]. In addition to viral infection, the Pelota protein has been implicated in rice bacterial blight resistance by activating the salicylic acid pathway [54], the key pathway for plant defence [7], demonstrating NGD might be involved in global plant pathogen response.

### Control of TEs

Almost all living organisms contain transposons in their genomes [30]. They are best characterized by a unique ability to change their location and replicate themselves within the genome, posing significant threat to genome integrity. To maintain genomic stability, TEs are epigenetically silenced. It is very well documented that a large portion of TE control results from DNA methylation and histone modifications [24]. In plants, these epigenetic silencing markers originate from processes such as RNA-directed DNA methylation (RdDM). Briefly, the RdDM cycle is initiated by DNA-DEPENDENT RNA POLYMERASE IV (pol IV), a plant-specific RNA polymerase [9]. Pol IV transcribes short RNAs from methylated TE loci. These RNAs are converted into double-stranded RNA (dsRNA) by RNA-DEPENDENT RNA POLYMERASE 2 (RDR2) that is anchored to pol IV. Subsequently, the endonuclease DICER-LIKE 3 (DCL3) cleaves the dsRNA into 24-nucleotide (nt) short interfering RNA (siRNA) prior to loading into ARGONAUTE 4 (AGO4). AGO4 delivers the 24-nt siRNA, along with DNA methyltransferases, to the scaffold RNAs transcribed by pol V, another plant-specific RNA polymerase, reinforcing the silent state of TEs and iterating the RdDM cycle [29, 30, 33, 55].

In addition to canonical RdDM described above, an alternative RDR6-mediated RdDM has previously been implicated in the silencing of reactivated TEs by targeting mRNAs derived from active TEs [5, 36]. In this pathway, RDR6 plays the same role as RDR2, but in the cytoplasm and on defective transcripts, for instance, uncapped, tail-less, or truncated mRNAs as substrates [1]. Although the mechanism that initiates replication of defective mRNA has not yet been described, it is likely linked to the localisation of RDR6 to cytoplasmic stress granules (SGs), involving the action of an RNA-binding protein, SUPPRESSOR OF GENE SILENCING 3 (SGS3) [16, 48]. Subsequent cleavage of dsRNA by DCL2/4 generates 21/22-nt siRNAs, which can both target TE RNAs for cleavage like miRNAs and induce *de novo* DNA methylation at the cognate loci [37].

As previously mentioned, RNA truncation is usually followed by RNA degradation through various RQC systems of the host. However, RNA cleavage at TE RNAs is instead associated with RDR6-mediated siRNA pathway, which is contrasted with the well-established mechanism suggesting that RQC overrides siRNA production to prevent trans-acting, indirect, and potentially adverse effects to endogenous mRNAs [6, 25]. In fact, such feature of favouring siRNA production by TEs is beneficial to the host because it allows for perpetual and stable control of TEs. Despite its critical importance, the mechanisms determining the RNA fates to either RQC or siRNA have long been a major knowledge gap in the field.

### Ribosome stalling triggers RDR6-RdDM

Previous attempts to address this fundamental question include the study by Baeg et al., which suggested that RDR6 specifically targets transcripts that are incomplete or truncated [1]. Moreover, RDR6 and SGS3, an RNA-binding protein essential for RDR6-RdDM, have been shown to localize in cytoplasmic SGs [16], a non-membranous subcellular compartment that is formed upon stress and stores various RNAs and proteins.

It has been known for years that many TEs are composed of sub-optimal codons, codons that are rarely used in transcripts or associated with poor translational activities [4, 16], although its biological implication has been obscure. Owing to this sequence characteristics, TE RNAs often accumulate stacks of stalled ribosomes, potentially underpinning the mechanism by which they are detected as separate from genic mRNAs. In our previous work, this hypothesis was empirically tested in rice and Arabidopsis, demonstrating that sub-optimal codons cause ribosome stalling and subsequently inefficient translation [16]. Reduced translational activity of TE RNAs nicely accounts for the two important criteria for siRNA biogenesis previously mentioned. That is, on one hand, ribosome stalling triggers RNA cleavage, a minimal and essential prerequisite for RDR6 action, and on another hand, reduced translation leads to RNA localization to SGs. Therefore, being sub-optimal in sequence and weak in translation, TE RNAs can be preferentially targeted to the siRNA production pathway by cytologically locating to where RDR6 is present and biochemically becoming competent to RDR6 action.

Ribosome stalling-triggered siRNA biogenesis was further supported by a more recent paper by Oberlin et al. They utilised the TE *EVADE* (*EVD*) from the Ty1/Copia family, reactivating it in Arabidopsis using the DNA methylation-deficient mutant, *decrease in DNA methylation* (*ddm1*). The proposed mechanism for regulation centred around short subgenomic mRNA (shGAG), produced in abundance from *EVD*, resulting in 21/22-nt siRNAs. The shGAG transcripts production coincides with ribosome stalling and mRNA cleavage resulting in 5’-OH ends. The presence of 5’-OH are a strong indicator of NGD involvement as a result of ribosome stalling on shGAG RNA [35]. Interestingly, ribosome stalling was also established in the *rdr6* mutant, suggesting that ribosome stalling is inherently induced regardless of RDR6 recruitment.

In order for an NGD-induced RNA ends to be processed and completely degraded by RNA decay, the 5’-OH RNA end should be phosphorylated, which in yeast is catalysed by Trl1. Trl1 is highly conserved across eukaryotic organisms, and Arabidopsis seems to have one copy of it in the genome, however, its function in TE-siRNA production is entirely unknown. It is nonetheless a compelling hypothesis that Trl1 might be the key fate-determining factor governing the entry to either RQC or siRNA biogenesis. Although it is possible these pathways are not mutually exclusive, exonuclease-mediated degradation of NGD-cleaved products after phosphorylation by Trl1 would likely deprive RDR6 of substrate transcripts. Furthermore, canonically the 40 S ribosomal subunit remains attached to NGD-cleaved mRNA unless recycled by Tma20/Tma22 [53], however, it is unclear if the 40 S subunit plays any roles in RDR6 recruitment and siRNA biogenesis.

Although NGD factors have not yet tested directly in the process of TE-derived siRNA production in Arabidopsis, Pelota1 has been previously shown to suppress the production of miRNA-triggered and phased secondary siRNAs [51], which is again reminiscent of dominant effect of RQC over siRNA production. Taken together, NGD likely plays an important role in TE regulation and the distinction between genic mRNA and TE mRNA.

### Transcriptome-wide NGD landscape

So far, we have highlighted several recent findings regarding NGD and its biological functions in plants. Apart from these genetic approaches, biochemical assays have been employed to unravel the NGD occurrence within plant cells. For example, the exploration of 5’-truncated mRNA ends by Hou et al. uncovered mRNA degradation fragments protected by stacks of stalled ribosomes [12]. In addition, using parallel analysis of RNA ends (PARE)-seq and RNA degradome data, 5’-truncated ends associated with upstream open reading frames (uORFs) and coding sequences were identified in Arabidopsis [11]. uORFs are regulatory sequences located in the 5’UTR of an mRNA, associated with reduced gene expression via their poor translational efficiency [2]. These studies support this hypothesis by revealing stalled ribosomes on uORFs as well as initiating mRNA decay.

**Fig. 1:**
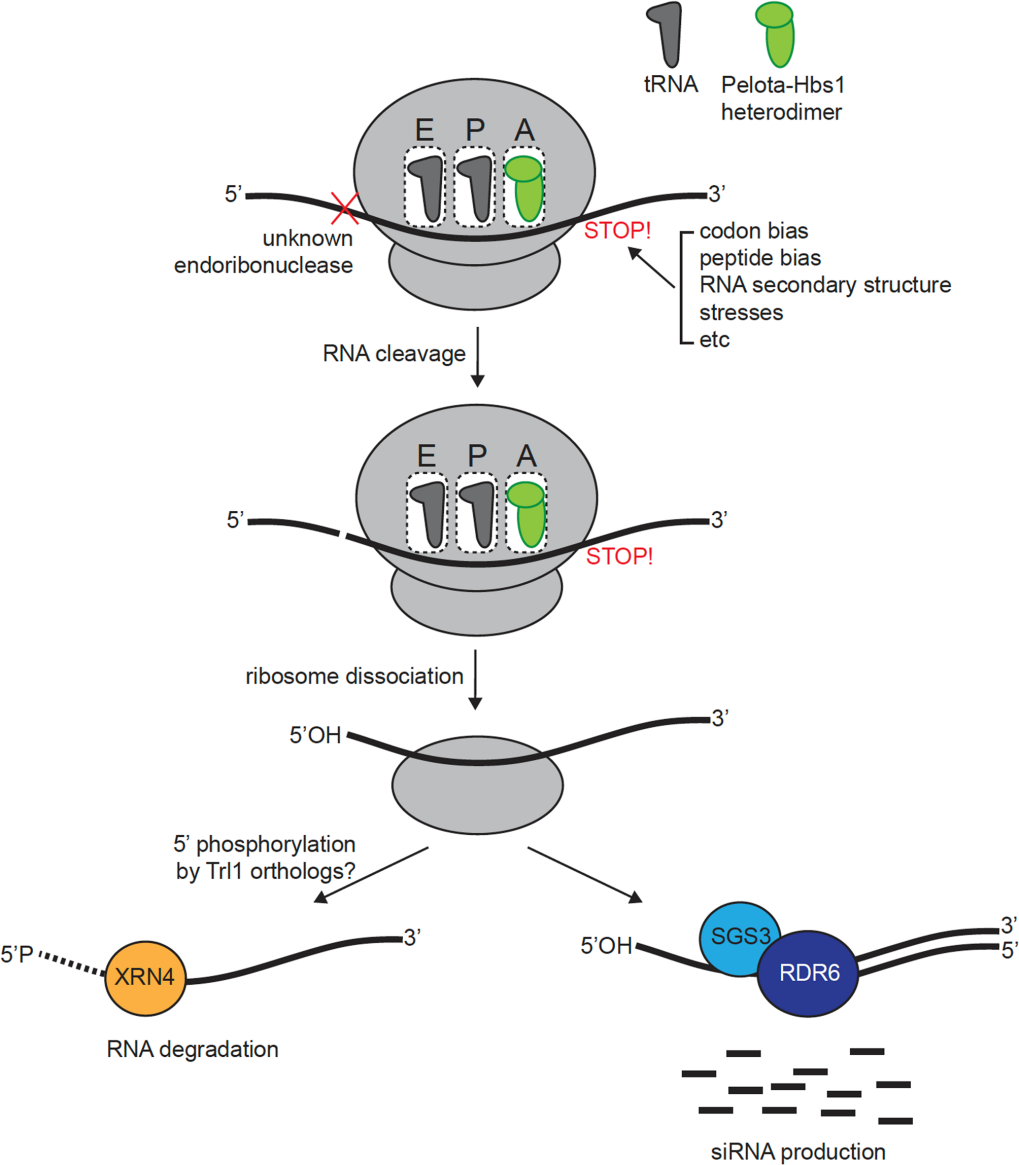
Ribosome stalling, RNA decay, and RDR6-RdDM pathway. Ribosome stalling can be triggered by various factors, including codon and peptide biases, RNA secondary structures, and environmental stress. When stalling occurs, the ribosomal A site is occupied by a Pelota-Hbs1 heterodimer, initiating the NGD pathway. This pathway involves endonucleolytic cleavage of the mRNA by an as-yet unidentified endoribonuclease, followed by ribosome disassembly. The cleavage produces RNA fragments with a 5’OH end, which must be phosphorylated prior to degradation by 5’−3’ exoribonucleases. In yeast, this phosphorylation is carried out by the Trl1 kinase, but the equivalent enzyme in plants remains unknown. Notably, the 5’OH RNA fragments are believed to be preferential substrates for RDR6, promoting siRNA production

Despite producing valuable data about the nature of ribosome stalling and truncated mRNA, a potential limitation of these studies is that PARE-seq rely on a 5’ phosphate group on the ends of the truncated mRNA for sequencing [11]. A proposed mechanism for endoribonucleolytic cleavage triggered by NGD suggests that Dom34-Hbs1-mediated cleavage results in truncated mRNA with a hydroxyl group at the 5’ end. Therefore, the captured mRNA 5’ ends, associated with ribosome arrest, are supposedly not the direct products of NGD, leaving the plant NGD landscape still ambiguous (Fig. 1).

In budding yeast, mapping of 5’-OH ends produced by NGD was attempted by Peach et al. utilising the RtcB enzyme extracted from *E. coli*, which catalyses phosphorylation of 5’-OH ends of RNA [32, 38]. Once 5’-OH ends have been phosphorylated, they are then available for adapter ligation and sequencing. This approach facilitates direct analysis of 5’-OH ends rather than having to analyse a phosphorylated intermediate. 5’-OH ends were identified in high frequencies upstream of consecutive glutamate and lysine or arginine residues. As a result, they suggested the mRNA decay events were related to the poly-electrostatic amino acid stretches clashing with the negatively charged ribosome exit tunnel, leading to ribosome stalling and NGD [43, 44].

### Summary and future perspectives

The aim of this paper is to establish the current research landscape and existing data that supports the involvement of the NGD RQC pathway in diverse biological processes, particularly in the control of invasive genetic elements. A key feature in TE RNA truncation by NGD is that it is funnelled to the siRNA pathway rather than RNA decay. A hitherto unknown factor that is able to phosphorylate the NGD-induced 5’-OH RNA ends in plants, acting like Trl1 in yeast, should supposedly possess differing activity between genic and TE RNAs. Such differentiation might be achieved by specific localization of this hypothetical protein, presumably in P body as it is the main cytoplasmic site where RNA degradation occurs. Identifying this kinase and characterizing its mode of action in plants will be a critical next step to be investigated. Furthermore, the roles of factors such as Hel2 (ubiquitination of 40 S subunit) and Cue2 (RNA cleavage at ribosome stalled site) have not yet been studied in plants. Identifying and characterizing the functional orthologs of these factors in plants could elucidate further how NGD products are pipelined into RNA decay or siRNA production.

Although the ribosome arrest-associated RNA cleavage sites have been known in plants, the precise and direct NGD targets are yet to be determined. A detailed map of NGD landscape and an integrative analysis combining various RNA features, such as RNA secondary structure and codon biases [39] will enable us to peer at the local trigger of NGD. Therefore, a similar approach employed by Peach et al. will be warranted to address this point. Moreover, most cases introduced in this review on the involvement of NGD are based on the studies that used the mutants for NGD factors, and no direct evidence was ever shown for ribosome stalling and NGD-mediated RNA truncation. The new method capturing 5’OH-ends in plants will help answer these questions as well.

The roles of NGD extend beyond virus and transposon proliferation, and literatures suggest that it plays a role in controlling endogenous mRNAs. For example, loss of function of *Pelota1* partially rescued the phenotype of reduced growth and delayed bolting in Arabidopsis 3′-phosphoinositide-dependent protein kinase1 (PDK1) mutants, *pdk1.1* and *pdk1.2* [20]. Moreover, in the study of Guo and Gregory, they observed reduced germination rates in *Pelota1* and *Hbs1* null mutants (Guo & Gregory, 2023), though the mechanistic details remain to be elucidated. In addition, considering that exposure to abiotic stresses such as, heat, salinity and drought can result in ribosome stalling in plants [45] (Imamichi et al., 2024), further research may reveal the roles of NGD in ribosome rescue as key for plant survival under stress conditions and resuming normal translation when stress conditions alleviate.

Moreover, the addition of small ubiquitin-like modifier (SUMO) protein to Pelota was suggested to be crucial for Hbs1 recruitment [10]. This identifies blocking Pelota SUMOylation as a potential target for suppressing Pelota function and therefore conferring viral resistance. This concept has begun to be explored by Li et al. who proposed the viral RNA-dependent RNA polymerase NIb as a SUMOylation decoy to competitively inhibit Pelota [22]. This represents a potential biological target for therapeutics leading to viral resistance in plants.

Finally, across all domains of life, organisms have evolved innate mechanisms to recognize and neutralize foreign genetic material. In bacteria, restriction-modification systems use host-specific DNA methylation patterns to distinguish self from non-self DNA, allowing unmethylated foreign DNA to be selectively degraded by restriction endonucleases [42]. In eukaryotes, analogous innate systems include cytosolic DNA sensors such as the cGAS (cyclic GMP-AMP synthase)-STING (Stimulator of Interferon Genes) pathway in animals, which detects aberrant double-stranded DNA that is normally absent from the cytoplasm, and triggers type I interferon responses [46]. In addition to DNA-based mechanisms, plants rely on RNA-mediated pathways that process double-stranded RNA generated during viral infection or transposon reactivation into small RNAs that guide sequence-specific degradation. While the molecular basis for recognizing non-native RNA remains incompletely understood, emerging evidence points to NGD as critical for distinguishing self from non-self RNA, thereby contributing to the innate defense against invasive genetic elements.

## Data Availability

No datasets were generated or analysed during the current study.
